# Effect of annualized surgeon volume on major surgical complications for abdominal and laparoscopic radical hysterectomy for cervical cancer in China, 2004–2016: a retrospective cohort study

**DOI:** 10.1186/s12905-023-02213-6

**Published:** 2023-02-15

**Authors:** Cong Liang, Weili Li, Xiaoyun Liu, Hongwei Zhao, Lu Yin, Mingwei Li, Yu Guo, Jinghe Lang, Xiaonong Bin, Ping Liu, Chunlin Chen

**Affiliations:** 1 Department of Obstetrics and Gynecology, Nanfang Hospital, Southern Medical University, No. 1838 Guangzhou Avenue, Guangzhou, 510515 China; 2 Department of Gynecology, The Third Affiliated Hospital of Zunyi Medical University, Zunyi, China; 3 Department of Gynecology, Shanxi Provincial Cancer Hospital, Taiyuan, China; 4 Department of Obstetrics and Gynecology, the Jiangmen Central Hospital of SUN YAT-SEN University, Jiangmen, China; 5 Department of Gynecology, Anyang Tumor Hospital, Anyang, China; 6 Department of Obstetrics and Gynecology, Peking Union Medical College Hospital, Peking Union Medical College & Chinese Academy of Medical Science, Beijing, China; 7 Department of Epidemiology, College of Public Health, Guangzhou Medical University, Guangzhou, China

**Keywords:** Cervical cancer, Radical hysterectomy, Cervical cancer, Major complications

## Abstract

**Background:**

Previous studies have suggested that higher surgeon volume leads to improved perioperative outcomes for oncologic surgery; however, the effect of surgeon volumes on surgical outcomes might differ according to the surgical approach used. This paper attempts to evaluate the effect of surgeon volume on complications or cervical cancer in an abdominal radical hysterectomy (ARH) cohort and laparoscopic radical hysterectomy (LRH) cohort.

**Methods:**

We conducted a population-based retrospective study using the Major Surgical Complications of Cervical Cancer in China (MSCCCC) database to analyse patients who underwent radical hysterectomy (RH) from 2004 to 2016 at 42 hospitals. We estimated the annualized surgeon volumes in the ARH cohort and in the LRH cohort separately. The effect of the surgeon volume of ARH or LRH on surgical complications was examined using multivariable logistic regression models.

**Results:**

In total, 22,684 patients who underwent RH for cervical cancer were identified. In the abdominal surgery cohort, the mean surgeon case volume increased from 2004 to 2013 (3.5 to 8.7 cases) and then decreased from 2013 to 2016 (8.7 to 4.9 cases). The mean surgeon case volume number of surgeons performing LRH increased from 1 to 12.1 cases between 2004 and 2016 (*P* < 0.01). In the abdominal surgery cohort, patients treated by intermediate-volume surgeons were more likely to experience postoperative complications (OR = 1.55, 95% CI = 1.11–2.15) than those treated by high-volume surgeons. In the laparoscopic surgery cohort, surgeon volume did not appear to influence the incidence of intraoperative or postoperative complications (*P* = 0.46; *P* = 0.13).

**Conclusions:**

The performance of ARH by intermediate-volume surgeons is associated with an increased risk of postoperative complications. However, surgeon volume may have no effect on intraoperative or postoperative complications after LRH.

**Supplementary Information:**

The online version contains supplementary material available at 10.1186/s12905-023-02213-6.

## Introduction

Cervical cancer ranks fourth among the most frequently diagnosed cancer in women (570,000 cases) [1], and radical hysterectomy (RH) with bilateral pelvic lymph node dissection is the recommended surgical treatment for women with early-stage disease [2]. The guidelines from the National Comprehensive Cancer Network (NCCN), International Federation of Gynecology and Obstetrics (FIGO) and National Institute for Health and Care Excellence (NICE) state that “the standard approach for RH is with an open abdominal approach” [2–4]. Although patient-related factors and clinical factors, such as obesity, diabetes, FIGO stage, the lack of standard medical care, surgical approach and hospital volume, are known to influence major complications during RH, there is growing recognition that surgeon volume also affects the outcomes for RH [5–8].

Regarding cervical cancer, the current engagement literature is focused mostly on the effects of hospital volume on clinical outcomes, with less attention given to the effects of surgeon volume on perioperative outcomes. For patients with early-stage cervical cancer, surgery at high-volume centres is associated with lower reduced perioperative morbidity and improved survival, whereas for patients with locally advanced disease, hospital volume has a minimal impact on survival [9–11]. In addition to resources available at the hospital, the outcome of a surgical procedure may depend mainly on surgeon volume [12]. Accumulative evidence to date has suggested that higher surgeon volume leads to improved perioperative surgical outcomes for various cancer, such as oesophageal cancer, brain tumours, pancreatic cancer, bladder cancer, endometrial cancer, rectal cancer and lung cancer [13–19]. However, some scholars argue that surgeon volume is not associated with complications [20, 21]. Recognition of the volume-outcomes paradigm in high-risk cancer procedures has led to changes in practice. Consequently, some health systems are instituting a minimum surgeon volume standard for high-risk surgery, such as oesophagectomy [19]. Wright et al. demonstrated that women treated by high-volume surgeons had fewer postoperative medical complications and lower transfusion requirements after abdominal RH (ARH) [22]. To date, few studies have investigated the association between surgeon volume and outcomes of RH in a cohort of abdominal cases and laparoscopic cases, respectively. In addition, there is a paucity of data on surgeon volume in Asian populations.

The objective of this population-based analysis is to explore the association between annualized surgeon volume and complications in an ARH cohort and a laparoscopic radical hysterectomy (LRH) cohort, respectively.

## Methods

### Data source

Data from the Major Surgical Complications of Cervical Cancer in China (MSCCCC) project database were utilized. The MSCCCC database is a multicentre retrospective database established to measure surgical quality. Approximately 97.5% of cases from the database could be matched to the cases in the Chinese Clinical Cervical Cancer (FOUR-C) project (http://www.chictr.org.cn/index.aspx, ChiCTR1800017778). The MSCCCC database gathers hospitalization information for 36,543 patients from 42 hospitals in 14 provinces of China from 2004 to 2016 (updated June 2020). The 42 hospitals consist of 32 general hospitals, 4 cancer centres and 6 women and children’s hospitals (W&C hospitals) [23].

By using the discharge diagnosis of “cervical cancer” as the keyword or the International Classification of Diseases Tenth Revision code C53.9 for computerized search, specially-trained gynaecologists abstracted data from medical records and the hospital information system. The documentation used for data extraction on complications included inpatient medical records for surgical treatment, postoperative adjuvant therapy records within 6 months, outpatient records, and readmission to the another department for complication treatment within 2 years [23]. The MSCCCC database collects data on patient demographics, clinical characteristics, and hospital factors. After completion of double data entry, data checking was carried out by two independent gynaecologists to eliminate input errors and logic errors. Data masking was used to protect patient privacy. Ethical approval was obtained from the Institutional Ethics Committee of Southern Medical University Nanfang Hospital (NFEC-2017–135).

### Cohort identification

Women who underwent RH for cervical cancer between 2004 and 2016 were analysed. The inclusion criteria were as follows: (1) the patient had been diagnosed with stage IA1 with LVSI (lymphovascular space invasion) to stage IIB disease, according to the 2009 FIGO staging system; (2) the patient had undergone type B or C RH (Querleu and Morrow classification) [24] + pelvic lymphadenectomy (PLN) ± para-aortic lymphadenectomy (PALA); and (3) the patient had undergone ARH or LRH. RH is mentioned as a treatment option for stage IIB disease in the Japan Society of Gynaecologic Oncology guidelines 2017 [25]. Chemoradiotherapy and neoadjuvant chemotherapy (NACT) followed by RH are recommended for patients with stage IIB cervical cancer in the European guidelines [26]. As such, we also included patients with FIGO stage IIB.

The exclusion criteria were as follows: (1) the patient was diagnosed with cervical cancer during pregnancy, was diagnosed with incidental cervical cancer after extrafascial hysterectomy or had a prior history of other malignancies; (2) the patient had an unknown lymphadenectomy status or did not undergo lymphadenectomy; (3) the patient underwent laparoscopic-assisted radical vaginal hysterectomy or robot-assisted RH; or (4) the patient had missing surgeon data or a missing date of operation.

### Clinical and demographic characteristics

The demographic characteristics included age, year of surgery, urban‒rural distribution, mode of delivery, and comorbidities. Clinical characteristics that were analysed included FIGO stage, gross type of tumour, histological type, preoperative anticancer treatment, hysterectomy type and lymph node dissection. Other operation details included operative time and estimated blood loss.

The hospitals where patients were treated were characterized based on hospital function (general hospital, cancer centre, or W&C hospital), region of the country (north, south, central, east, southwest, northwest or northeast), and city scale (first-tier, second-tier, and third-tier or below). The levels of urban economic development were as follows: first-tier cities > second-tier cities > third-tier cities.

### Surgeon volume

Surgeons were sequentially numbered. Then we calculated the total volume for each surgeon, and the annualized surgeon volumes was calculated as the total number of procedures that the surgeon performed divided by the number of years in which an individual surgeon contributed to at least one RH [17, 22, 27]. Patients were stratified into two cohorts based on the hysterectomy approach: ARH group or LRH group. Surgeon volume cut-off points were then selected to divide patients into approximately equal tertiles. Abdominal surgeon cut-off points were as follows: low volume (≤ 8.1 procedures per year), intermediate volume (8.2–16.9 procedures per year), and high volume (> 16.9 procedures per year). Laparoscopic surgeon cut-off points were: low volume (≤ 11.0 procedures per year), intermediate volume (11.1–20.0 procedures per year), and high volume (> 20.0 procedures per year).

### Outcomes

Complications were divided into intraoperative complications and postoperative complications. Intraoperative complications, which included ureteral injury, bladder injury, bowel injury, vascular injury, obturator nerve injury, and stomach injury, were recorded. Postoperative complications included bowel obstruction, pelvic haematoma, haemorrhage, vesicovaginal fistula, ureterovaginal fistula, ureteral fistula, rectovaginal fistula, venous thromboembolism and chylous leakage. We also recorded deaths from surgical complications.

### Statistical analysis

The relationship between the number of surgeons and year was assessed by a nonparametric Spearman correlation test. Due to discontinuous data, one of the hospitals was not included in the graphs. Frequency distributions between categorical variables were compared using χ2 (Bonferroni corrected, if required) or Fisher’s exact test, and continuous variables were compared using one-way analysis of variance. The median and interquartile range (IQR) of surgeon volume were also reported for each tertile. Binary logistic regression models were used to determine predictors of treatment by the intermediate and high volume (highest 2/3 volume) surgeons. Demographic, clinical, and hospital characteristics constituted independent variables. To examine the association between surgeon volume and outcome, we built binary logistic regression models including surgeon volume while adjusting for the other variables described above. The results are reported as odds ratios (ORs) and 95% confidence intervals (CIs). A value of *P* < 0.05 was considered statistically significant. All analyses were performed with the SPSS 23.0 statistical software package (SPSS, Inc., Chicago, IL, USA).

## Results

### Trends in the number of surgeons and patients

**Fig. 1:**
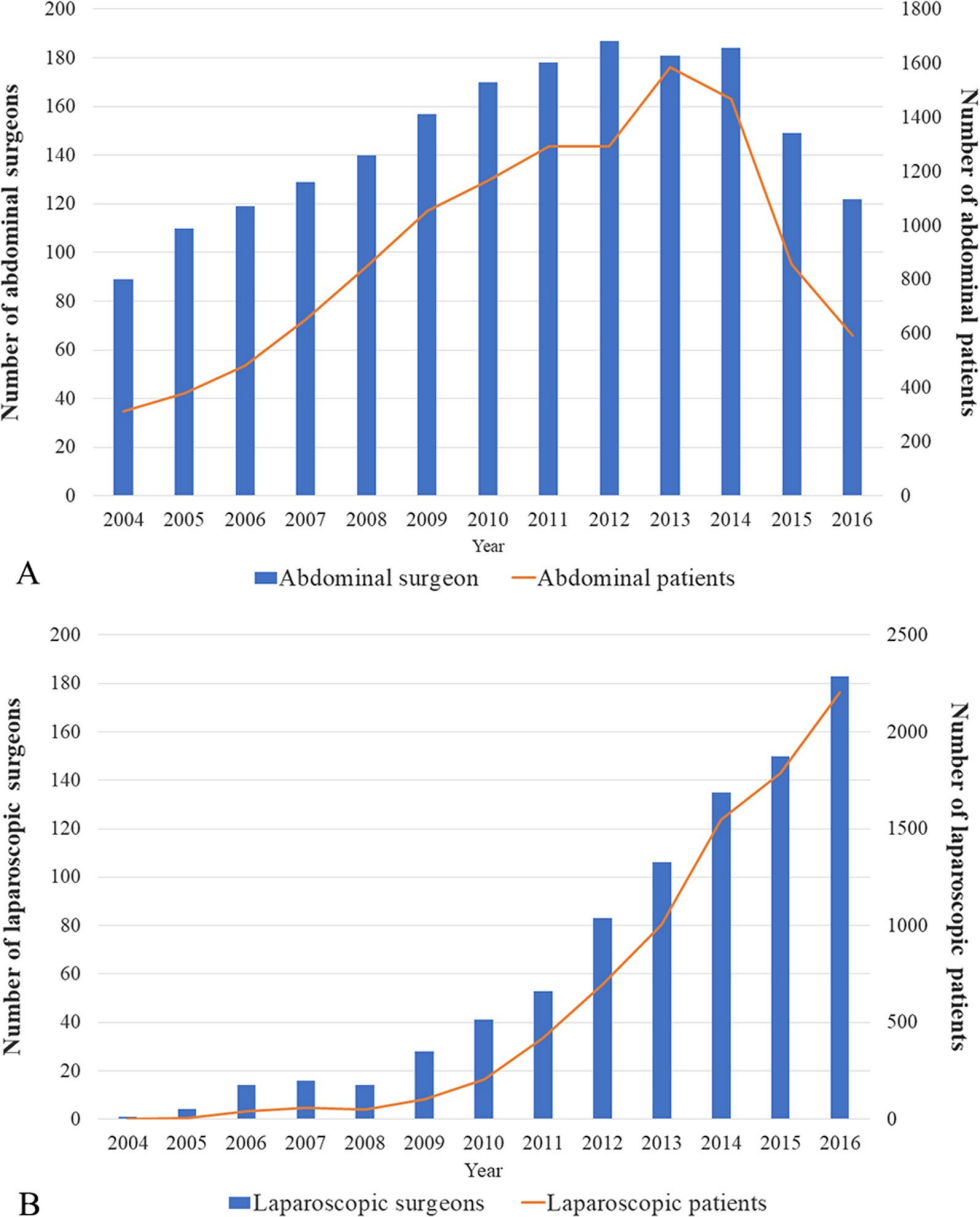
Numbers of patients and surgeons by year in the abdominal surgery cohort (**A**); Numbers of patients and surgeons by year in laparoscopic surgery cohort (**B**)

We identified a total of 22,684 patients, including 14,536 (64.1%) patients who underwent ARH and 8148 (35.9%) patients who underwent LRH (Table 1). The number of surgeons performing ARH each year increased from 89 surgeons who operated on 313 patients in 2004 to 187 surgeons who operated on 1,294 patients in 2012 (*P* < 0.01, r = 0.99). However, the number of abdominal surgeons decreased to 122 surgeons with 594 patients in 2016 (*P* = 0.20, r = − 0.80). The mean surgeon case volume increased from 3.5 cases in 2004 to 8.7 cases in 2013 and then decreased to 4.9 cases in 2016 (Fig. 1A). The number of surgeons performing LRH increased from 1 surgeon with 1 patient in 2004 to 183 surgeons who operated on 2,206 patients in 2016 (*P* < 0.01, r = 0.99). The mean number of surgeons performing LRH increased from 1 to 12.1 cases between 2004 and 2016 (Fig. 1B).

**Table 1:** Schema of patient selection

Variable	No. of patients
1.All patients diagnosed with cervical cancers, 2004–2016	36,543
2. Type B or C radical hysterectomy	31,581
3. Pelvic lymphadenectomy ± para-aortic lymphadenectomy	30,641
4. 2009 FIGO stage IA1 with LVSI to IIB	24,447
5. No pregnancy, incidental cervical cancer or previous malignant disease	24,350
6. Abdominal surgery or total laparoscopic surgery	23,409
7. Missing information of surgeon or date of surgery	22,684

### Characteristics of the cohort

In the ARH cohort, the median volume of surgeons in the low-volume group was two (IQR 1.0–3.5) per year and rose to 30.9 (IQR 21.9–42.1) in the high volume group. Similarly, in the LRH cohort, the median volume of surgeons in the low-volume group was 2.3 (IQR 1.0–5.0) and increased to 27.8 (IQR 21.6–30.1) in the high-volume group (Table 2). In both the ARH and LRH cohorts, the operation time and bleeding loss for patients in the high-volume surgeon group were significantly lower than those for patients in the intermediate-volume and low-volume groups (*P* < 0.001) (Table 2).

**Table 2:** Demographic and clinical characteristics of the abdominal and laparoscopic cohorts stratified by surgeon volume

Characteristic	Abdominal surgeon volume						*P* value	Laparoscopic surgeon volume						*P* value
	Low		Intermediate		High			Low		Intermediate		High		
*Patients*	4837	33.3	4913	33.8	4786	32.9		2642	32.4	2637	32.4	2869	35.2	
*Surgeons*	361		44		22			212		33		18		
*Annualized hospital volume*	2.0 (1.0–3.5)		10.8 (9.1–12.5)		30.9 (21.9–42.1)			2.3 (1.0–5.0)		13.5 (12.3–16.9)		27.8 (21.6–30.1)		
*Range*	1.0	8.1	8.2	16.9	17.0	129.4		1.0	11.0	11.1	20.0	20.1	51.7	
*Age at surgery, years*														
≥ 60	444	9.2	515	10.5	798	16.7	< 0.001	257	9.7	299	11.3	346	12.1	0.02
< 60	4393	90.8	4398	89.5	3988	83.3		2385	90.3	2338	88.7	2523	87.9	
*Year of diagnosis*														
2004–2009	1736	35.9	1748	35.6	245	5.1	< 0.001	149	5.6	46	1.7	59	2.1	< 0.001
2010–2016	3101	64.1	3165	64.4	4541	94.9		2493	94.4	2591	98.3	2810	97.9	
*Urban‒rural distribution*														
Rural	2316	47.9	2988	60.8	3098	64.7	< 0.001	1572	59.5	1450	55.0	1638	57.1	< 0.001
Urban	1423	29.4	1539	31.3	1004	21.0		739	28.0	959	36.4	1055	36.8	
Unknown	1098	22.7	386	7.9	684	14.3		331	12.5	228	8.6	176	6.1	
*Hospital function*														
General hospital	3451	71.3	2893	58.9	641	13.4	< 0.001	1998	75.7	2097	79.5	1526	53.2	< 0.001
Cancer centre	1088	22.5	1626	33.1	4145	86.6		450	17.0	386	14.6	1223	42.6	
W&C centre	298	6.2	394	8.0	0	0.0		194	7.3	154	5.8	120	4.2	
*Region*														
North	724	15.0	1292	26.3	2784	58.2	< 0.001	589	22.3	220	8.3	605	21.1	< 0.001
South	1380	28.5	1095	22.3	531	11.1		688	26.1	427	16.2	120	4.2	
Central	618	12.8	410	8.3	1169	24.4		350	13.2	700	26.5	231	8.1	
East	1490	30.8	1013	20.6	302	6.3		752	28.5	396	15.0	0	0.0	
Southwest	307	6.3	335	6.8	0	0.0		91	3.4	507	19.2	1808	63.0	
Northwest	158	3.3	668	13.6	0	0.0		161	6.1	323	12.2	105	3.7	
Northeast	160	3.3	100	2.0	0	0.0		11	0.4	64	2.4	0	0.0	
*City scale*														
First-tier	773	16.0	745	15.2	529	11.1	< 0.001	560	21.2	299	11.3	0	0.0	< 0.001
Second-tier	2458	50.8	2885	58.7	3986	83.3		1471	55.7	1916	72.7	2362	82.3	
Third-tier	1606	33.2	1283	26.1	271	5.7		611	23.1	422	16.0	507	17.7	
*Mode of delivery*														
No delivery	49	1.0	57	1.2	22	0.5	< 0.001	23	0.9	23	0.9	36	1.3	< 0.001
Vaginal delivery	3684	76.2	4026	81.9	4059	84.8		2114	80.0	2157	81.8	2541	88.6	
Caesarean delivery	253	5.2	291	5.9	221	4.6		194	7.3	243	9.2	261	9.1	
Unknown	851	17.6	539	11.0	484	10.1		311	11.8	214	8.1	31	1.1	
*Comorbidity*														
No	4526	93.6	4592	93.5	4242	88.6	< 0.001	2425	91.8	2436	92.4	2611	91.0	0.18
Yes	311	6.4	321	6.5	544	11.4		217	8.2	201	7.6	258	9.0	
*FIGO stage*														
IA1 + IA2	133	2.7	76	1.5	30	0.6	< 0.001	70	2.7	49	1.9	45	1.6	< 0.001
IB1	2288	47.3	2117	43.1	2282	47.7		1554	58.8	1323	50.2	1318	45.9	
IB2	656	13.6	690	14.0	531	11.1		289	10.9	295	11.2	328	11.4	
IIA1	679	14.0	680	13.8	1278	26.7		350	13.2	329	12.5	586	20.4	
IIA2	297	6.1	324	6.6	478	10.0		123	4.7	176	6.7	270	9.4	
IIB	784	16.2	1026	20.9	187	3.9		256	9.7	465	17.6	322	11.2	
*Gross type*														
Exophytic	2585	53.4	2854	58.1	2058	43.0	< 0.001	1287	48.7	1357	51.5	1540	53.7	< 0.001
Endophytic	276	5.7	316	6.4	1528	31.9		128	4.8	73	2.8	185	6.4	
Ulcerated	900	18.6	790	16.1	550	11.5		461	17.4	467	17.7	699	24.4	
Endocervical	105	2.2	64	1.3	29	0.6		45	1.7	39	1.5	44	1.5	
After conization	132	2.7	106	2.2	74	1.5		80	3.0	97	3.7	102	3.6	
Preclinical carcinoma	342	7.1	414	8.4	178	3.7		298	11.3	256	9.7	140	4.9	
Unknown	497	10.3	369	7.5	369	7.7		343	13.0	348	13.2	159	5.5	
*Histological types*														
Squamous cell	4178	86.4	4326	88.1	4301	89.9	< 0.001	2252	85.2	2223	84.3	2481	86.5	0.008
Adenocarcinoma	450	9.3	397	8.1	294	6.1		262	9.9	293	11.1	272	9.5	
Adenosquamous	113	2.3	92	1.9	94	2.0		45	1.7	54	2.0	49	1.7	
Other subtypes	81	1.7	90	1.8	89	1.9		48	1.8	43	1.6	56	2.0	
Unknown	15	0.3	8	0.2	8	0.2		35	1.3	24	0.9	11	0.4	
*Preoperative treatment*														
No received	3457	71.5	3565	72.6	3911	81.7	< 0.001	2038	77.1	2098	79.6	2164	75.4	< 0.001
Neoadjuvant chemotherapy	1243	25.7	970	19.7	594	12.4		557	21.1	449	17.0	667	23.2	
Preoperative radiotherapy	137	2.8	378	7.7	281	5.9		47	1.8	90	3.4	38	1.3	
*Lymph node dissection*														
PLA	4651	96.2	4604	93.7	4735	98.9	< 0.001	2269	85.9	2028	76.9	1905	66.4	< 0.001
PLA + PALA	186	3.8	309	6.3	51	1.1		373	14.1	609	23.1	964	33.6	
*Hysterectomy types*														
Type B	2852	59.0	2140	43.6	3340	69.8	< 0.001	1047	39.6	592	22.4	242	8.4	< 0.001
Type C2	1962	40.6	2719	55.3	1424	29.7		1558	59.0	1991	75.5	2616	91.2	
Type C1	23	0.5	54	1.1	22	0.5		37	1.4	54	2.0	11	0.4	
*Operation time (min)*	232.8 ± 64.8		205.0 ± 55.4		164.7 ± 52.8		< 0.001	259.6 ± 77.1		234.4 ± 70.8		209.6 ± 64.6		< 0.001
*Blood loss (ml)*	499.2 ± 363.1		470.3 ± 375.14		241.0 ± 211.6		< 0.001	216.2 ± 305.7		193.0 ± 188.6		185.1 ± 170.1		< 0.001

*PLA* pelvic lymphadenectomy, *PALA* para-aortic lymphadenectomy

In the multivariable model of the ARH cohort, patients diagnosed in later years, patients living in rural areas, patients with a higher tumour stage (except stage IIB), patients with the endophytic or preclinical gross type, patients with preoperative radiotherapy, patients undergoing type C1 or C2 hysterectomy, patients undergoing surgery in a W&C hospital or cancer centre, and patients undergoing surgery in a hospital of a first-tier city were more likely to be treated by high-volume or intermediate-volume surgeons (*P* < 0.05) (Table 3).

**Table 3:** Predictors of high- and intermediate-volume surgeons

	Abdominal cohort		Laparoscopic cohort	
	OR (95% CI)	P value	OR (95% CI)	P value
*Age at surgery, years*		0.15		0.002
≥ 60	Ref		Ref	
< 60	0.91 (0.79–1.04)	0.15	0.74 (0.61–0.90)	0.002
*Year of diagnosis*		< 0.001		0.02
2004–2009	Ref		Ref	
2010–2016	1.55 (1.41–1.69)	< 0.001	1.47 (1.07–2.02)	0.02
*Urban–rural distribution*		< 0.001		0.08
Rural	Ref		Ref	
Urban	0.87 (0.80–0.96)	0.005	1.11 (0.97–1.27)	0.13
Unknown	0.51 (0.45–0.58)	< 0.001	0.87 (0.71–1.07)	0.20
*Hospital function*		< 0.001		< 0.001
General hospital	Ref		Ref	
Cancer center	3.45 (3.07–3.87)	< 0.001	1.83 (1.49–2.24)	< 0.001
W&C center	1.55 (1.27–1.89)	< 0.001	0.26 (0.20 -0.35)	< 0.001
*Region*		< 0.001		< 0.001
North	Ref		Ref	
South	0.45 (0.38–0.54)	< 0.001	4.91 (3.66–6.59)	< 0.001
Central	0.91 (0.78–1.06)	0.23	2.12 (1.68–2.68)	< 0.001
East	0.37 (0.32–0.42)	< 0.001	0.36 (0.28–0.45)	< 0.001
Southwest	1.05 (0.84–1.31)	0.70	82.00 (57.57–116.82)	< 0.001
Northwest	3.31 (2.56–4.28)	< 0.001	1.29 (0.88–1.88)	0.19
Northeast	0.22 (0.16–0.29)	< 0.001	3.36 (1.71–6.59)	< 0.001
*City scale*		< 0.001		< 0.001
First-tier	Ref		Ref	
Second-tier	0.70 (0.59–0.85)	< 0.001	8.74 (6.31–12.10)	< 0.001
Third-tier	0.36 (0.31–0.43)	< 0.001	1.18 (0.89–1.56)	0.26
*Mode of delivery*		< 0.001		0.004
No delivery	1.00 (0.68–1.48)	0.99	1.06 (0.57–1.97)	0.85
Vaginal delivery	Ref		Ref	
Cesarean delivery	1.06 (0.89–1.27)	0.54	1.35 (1.10–1.68)	0.005
Unknown	0.66 (0.59–0.74)	< 0.001	1.34 (1.07–1.67)	0.01
*Comorbidity*		0.41		0.43
No	Ref		Ref	
Yes	0.93 (0.79–1.10)	0.41	1.09 (0.88–1.35)	0.43
*FIGO stage*		< 0.001		< 0.001
IA + IB1	Ref		Ref	
IB2	1.08 (0.94–1.23)	0.28	1.26 (1.02–1.57)	0.03
IIA1	1.28 (1.13–1.44)	< 0.001	1.23 (1.02–1.48)	0.02
IIA2	1.28 (1.07–1.52)	0.006	2.26 (1.70–3.01)	< 0.001
IIB	0.75 (0.63–0.88)	< 0.001	1.60 (1.20–2.14)	0.002
*Gross type*		< 0.001		0.009
Exophytic	Ref		Ref	
Endophytic	2.09 (1.78–2.45)	< 0.001	0.64 (0.48–0.86)	0.003
Ulcerated	0.87 (0.78–0.98)	0.02	1.16 (0.99–1.38)	0.08
Endocervical	0.56 (0.40–0.77)	< 0.001	0.89 (0.56–1.42)	0.62
After conization	0.85 (0.66–1.10)	0.23	1.17 (0.84–1.65)	0.35
Preclinical carcinoma	1.39 (1.19–1.63)	< 0.001	0.88 (0.72–1.08)	0.22
Unknown	0.70 (0.61–0.81)	< 0.001	1.04 (0.86–1.26)	0.71
*Histological types*		0.021		0.96
Squamous cell	Ref		Ref	
Adenocarcinoma	0.89 (0.77–1.03)	0.18	1.06 (0.87–1.29)	0.54
Adenosquamous	0.82 (0.62–1.07)	0.15	1.00 (0.65–1.56)	0.97
Other subtypes	0.66 (0.49–0.90)	0.008	0.98 (0.64–1.48)	0.91
Unknown	0.70 (0.31–1.60)	0.40	0.88 (0.50–1.57)	0.66
*Preoperative treatment*		< 0.001		< 0.001
No received	Ref		Ref	
Neoadjuvant chemotherapy	0.81 (0.72–0.91)	0.001	0.69 (0.57–0.82)	< 0.001
Preoperative radiotherapy	1.92 (1.53–2.40)	< 0.001	0.85 (0.58–1.26)	0.42
*Lymph node dissection*		0.98		0.04
PLA	Ref		Ref	
PLA + PALA	1.00 (0.81–1.23)	0.98	1.19 (1.01–1.40)	0.04
*Hysterectomy types*		< 0.001		< 0.001
Type B	Ref		Ref	
Type C2	1.75 (1.59–1.93)	< 0.001	2.33 (1.98–2.75)	< 0.001
Type C1	2.73 (1.61–4.64)	< 0.001	3.60 (2.27–5.72)	< 0.001

*PLA* pelvic lymphadenectomy, *PALA* para-aortic lymphadenectomy

Similarly, in the multivariable model of the LRH cohort, age > 60 years, later year of diagnosis, previous caesarean section, FIGO stage IIA2 or IIB, PLN + PALA, and type C1 or C2 hysterectomy were associated with treatment by a high-volume or an intermediate-volume surgeon (*P* < 0.05). In addition, patients undergoing surgery in the cancer centre, patients undergoing surgery in the hospital of a second-tier city, and patients undergoing surgery in the hospital of the southwest area were more likely to be treated by intermediate- or high-volume surgeons (*P* < 0.05). Patients with the endophytic gross type, patients with neoadjuvant chemotherapy, and patients undergoing surgery in W&C hospitals were associated with treatment by a low-volume laparoscopic surgeon (*P* < 0.05) (Table 3).

### The impact of surgeon volume on complications

In the univariate analysis of the ARH group, the overall complication rates were 3.06% for women treated by low-volume surgeons, 3.42% for those treated by intermediate-volume surgeons, and 2.01% for those treated by high-volume surgeons (*P* < 0.001) (Table 4). Compared with patients treated by intermediate-volume surgeons, those operated on by high-volume surgeons had fewer postoperative complications (2.87% vs. 1.69%, *P* < 0.001), fewer ureteral injuries (0.45% vs. 0.16%, *P* = 0.045), fewer bowel obstructions (1.28% vs. 0.61%, *P* = 0.003), and fewer ureterovaginal fistulas (0.43% vs. 0.13%, *P* = 0.007). There were no significant differences in the frequencies of other complications (*P* < 0.05).

**Table 4:** Unadjusted complications associated with radical hysterectomy for cervical cancer stratified by surgeon volume

Outcome	Abdominal surgeon volume						*P* value	Laparoscopic surgeon volume						*P* value
	Low		Intermediate		High			Low		Intermediate		High		
	N	%	N	%	N	%		N	%	N	%	N	%	
Any 1 complication	148	3.06^a^	168	3.42^a^	96	2.01^b^	< 0.001	137	5.19	150	5.69	148	5.16	0.62
Intraoperative complication	27	0.56	32	0.65	16	0.33	0.08	39	1.48	24	0.91	44	1.53	0.09
Ureteral injury	15	0.31^ab^	22	0.45^a^	8	0.16^b^	0.045	28	1.06	14	0.53	24	0.84	0.10
Bladder injury	3	0.06	4	0.08	1	0.02	0.55	3	0.11	4	0.15	8	0.28	0.34
Bowel injury	0	0	2	0.04	0	0	0.33	1	0.04	1	0.04	4	0.14	0.38
Vascular injury	9	0.18	2	0.04	5	0.10	0.10	4	0.15	4	0.15	7	0.24	0.69
Obturator nerve injury	0	0	2	0.04	2	0.04	0.48	5	0.19	1	0.04	2	0.07	0.24
Stomach injury	0	0	0	0	0	–	–	1	0.04	0	0	0	0	0.65
Postoperative complication	122	2.52^a^	141	2.87^a^	81	1.69^b^	< 0.001	102	3.86	127	4.81	110	3.83	0.12
Bowel obstruction	46	0.95^ab^	63	1.28^a^	29	0.61^b^	0.003	18	0.68	18	0.68	21	0.73	0.97
Pelvic hematoma	1	0.02	1	0.02	0	0	0.99	0	0	2	0.08	0	0	0.11
Hemorrhage	3	0.06	3	0.06	1	0.02	0.71	5	0.19	3	0.11	5	0.17	0.83
Vesicovaginal fistula	8	0.16	9	0.18	6	0.13	0.81	19	0.72	17	0.64	24	0.84	0.71
Ureterovaginal fistula	9	0.19^ab^	21	0.43^a^	6	0.13^b^	0.007	27	1.02^a^	47	1.78^b^	31	1.08^a^	0.02
Rectovaginal fistula	1	0.02	2	0.04	1	0.02	0.99	3	0.11	3	0.11	3	0.10	0.99
Ureteral fistula	3	0.06	3	0.06	1	0.02	0.71	4	0.15	2	0.08	2	0.07	0.67
Venous thromboembolism	51	1.05	43	0.88	37	0.77	0.34	28	1.06	38	1.44	24	0.84	0.10
Chylous leakage	1	0.02	0	0	0	0	0.66	3	0.11	5	0.19	3	0.10	0.69
Other														
Death	0	0	0	0	2	0.04	0.11	0	0	1	0.04	1	0.03	0.77

Different letters on the shoulder mark indicate significant differences (*P* < 0.05), and the same letter or no letter indicates that the difference is not significant (*P* ≥ 0.05)

In the univariate analysis of the LRH group, patients treated by intermediate-volume surgeons had the highest ureterovaginal fistula rate (low vs. intermediate vs. high volume = 1.02% vs. 1.78% vs. 1.08%, *P* = 0.02). The frequencies of the other complications among laparoscopic surgery cases were similar across the three groups (*P* > 0.05) (Table 4).

In the multivariable analysis of the ARH cohort, patients treated by intermediate-volume surgeons were more likely to experience postoperative complications (OR = 1.55, 95% CI = 1.11–2.15), especially bowel obstruction (OR = 1.75, 95% CI = 1.05–2.94) and ureterovaginal fistula (OR = 3.47, 95% CI = 1.27–9.53), than those treated by high-volume surgeons (Table 5; Additional file 1: Table S1). The postoperative complication rate was higher in the low-volume surgeon group than in the high-volume surgeon group, and the difference approached significance (OR = 1.38, 95% CI = 0.97–1.96, *P* = 0.07). However, abdominal surgeon volume had no influence on overall complications or intraoperative complications (P > 0.05). In the multivariable analysis of the LRH cohort, surgeon volume had no effect on overall complications, intraoperative complications or postoperative complications (P > 0.05).

**Table 5:** Multivariable analysis of factors associated with complications

	Abdominal surgeon volume				*P* value	Laparoscopic surgeon volume				*P* value
	Low	Intermediate	High			Low	intermediate	High		
Any 1 complication	3.06%	3.42%	2.01%		0.10	5.19%	5.69%	5.16%		0.26
			1.20	0.87–1.66	0.27			1.14	0.82–1.58	0.43
			1.39	1.02–1.89	0.04			1.26	0.95–1.67	0.11
Intraoperative complication	0.56%	0.65%	0.33%		0.21	1.48%	0.91%	1.53%		0.46
			0.46	0.19–1.09	0.08			1.23	0.67–2.26	0.51
			0.58	0.25–1.38	0.22			0.86	0.48–1.54	0.61
Postoperative complication	2.52%	2.87%	1.69%		0.04	3.86%	4.81%	3.83%		0.13
			1.38	0.97–1.96	0.07			1.09	0.75–1.58	0.65
			1.55	1.11–2.15	0.01			1.34	0.98–1.83	0.07

The middle row for each complication class was adjusted for clinical and demographic factors, including age, year of diagnosis, urban–rural distribution, hospital function, region, city scale, mode of delivery, comorbidity, FIGO stage, gross type, histological type, preoperative treatment, lymph node dissection, and hysterectomy type reported, with the odds ratio (95% CI) of low vs. high volume. The bottom row for each complication class is adjusted for the factors mentioned above, with the odds ratio (95% CI) of intermediate vs. high volume

## Discussion

Our findings suggest that in the ARH cohort, postoperative complication rates were higher among intermediate-volume surgeons, while patients treated by high-volume surgeons had fewer postoperative complications. However, in the LRH cohort, annualized surgeon volume had no effect on intraoperative or postoperative complications.

The trends in the number of surgeons and mean surgeon volume over time differed between the two surgical approaches. From 2004 to 2016, the number of laparoscopic surgeons and mean surgeon case volume rose annually. This increasing acceptance of the laparoscopic technique could be due to the short-term benefits of LRH, including a more cosmetically pleasing incision, less bleeding, less postoperative pain, and faster postoperative recovery. However, the opposite trend was observed in the ARH cohort. The number of abdominal surgeons and mean surgeon case volume had been decreasing since 2013. In addition, it is noteworthy that categorical definitions of surgeon volume varied substantially among the different studies. In the study by Wright et al. [22], a high-volume surgeon and a low-volume surgeon were defined as a surgeon who performed more than 3.75 and less than 2.25 ARHs per year, respectively, but our definitions of high volume and low volume were greater than 16.9 and less than or equal to 8.1 ARHs per year, respectively. The surgeon volume gap between the two groups was much larger than that of the study by Wright et al. In addition, our results revealed a positive correlation between the number of surgeons and the number of patients. The increasing number of surgeons could effectively alleviate disease stress at the national level; however, additional investigations are needed to determine whether the regionalization of RH is occurring in China. Spontaneous regionalization of gynaecological malignancy procedures has occurred in the US. At the hospital level, Matsuo et al.’s analysis showed that 89 centres that performed at least one trachelectomy for cervical cancer per year and only 6 centres met the criteria for top-decile centres in the United States [9]. The threshold of any RH to have a minimum trachelectomy volume of 1 case a year was 7.8 cases per year. These findings imply that trachelectomy may have already been regionalized to hospitals with high RH volumes. At the surgeon level, the surgical treatment of an increasing number of patients with endometrial cancer has been limited to a smaller number of surgeons. The increased complexity of treatment has resulted in more patients being referred from general gynaecologists to gynaecologic oncology subspecialists [17].

Our data demonstrated that the association between surgeon volume and complications for cervical cancer was complex with an increased risk of postoperative complications among intermediate-volume surgeons but the lowest postoperative complication rates for the highest-volume surgeons in the ARH cohort. In the multivariable analysis of the ARH cohort, patients treated by intermediate-volume surgeons were more likely to experience postoperative complications (OR = 1.55, 95% CI = 1.11–2.15), especially bowel obstruction (OR = 1.75, 95% CI = 1.05–2.94) and ureterovaginal fistula (OR = 3.47, 95% CI = 1.27–9.53), than those treated by high-volume surgeons. In addition, the postoperative complication rate was higher in the low-volume surgeon group than in the high-volume surgeon group, and the difference approached significance (OR = 1.38, 95% CI = 0.97–1.96, *P* = 0.07). This observation resembles that of Wright et al. [22]. Their results showed that the perioperative complication rate was highest in the intermediate-volume surgeon group compared with the low-volume and high-volume surgeon groups (low vs. intermediate vs. high = 2.9% vs. 6.7% vs. 1.8%, *P* < 0.001). Additionally, compared with patients treated by low-volume surgeons, those operated on by high-volume surgeons had fewer medical complications and shorter lengths of stay [22]. However, no direct comparisons between the intermediate-volume surgeon group and other groups were performed in their study. Another study evaluating the influence of surgeon volume on morbidity with regard to hysterectomy reached the similar conclusions: outcomes are significantly worse among low-volume surgeons than among higher-volume surgeons for abdominal hysterectomy [28]. The majority of high-volume surgeons benefited from the effect of a sufficient learning curve and close cooperation with an experienced surgical team, which translates into a lower complication rate. One possible reason for the intermediate-volume surgeon group having the highest rate of postoperative complications could be that they were more lenient in their selection of patients. Patients treated by intermediate-volume surgeons had a higher frequency of FIGO stage IIB, more preoperative radiotherapy, and a higher frequency of PLA + PALA and type C2 hysterectomy. In the Laparoscopic Approach to Cervical Cancer (LACC) trial, ARH was associated with higher disease-free survival and overall survival rates than minimally invasive RH among women with early-stage cervical cancer [29]. The publication of the results of the LACC trial led to important changes in the management of cervical cancer patients worldwide [30]. After this publication, the number of patients treated with minimally invasive RH decreased from 64.9% to 30.4% [31]. ARH therefore has re-emerged as a mainstream treatment for cervical cancer, bringing attention to the effect of abdominal surgeon volume on the complication rates.

Despite the finding of poor survival with minimally invasive surgery (MIS) in LACC trail, some studies were still conducted to re-establish the role of MIS and select the appropriate patient for MIS, considering the tumour size and the histological type [32–35]. Thus, further exploration of the effect of surgeon volume on complications among patients who undergo LRH is also warranted. In our laparoscopy surgery cohort, surgeon volume had no significant effects on intraoperative complications or postoperative complications. It appears that the effects of surgeon volume on RH complications differ according to the surgical routes used. Similar results of endometrial cancer have reported that among patients who underwent abdominal hysterectomy for endometrial cancer, increased surgical volume was associated with reductions in perioperative surgical complications and medical complications [36]. However, during laparoscopic hysterectomy, the surgeon volume appeared to have little effect on perioperative morbidity for endometrial cancer [27]. A retrospective analysis of 1016 laparoscopic hysterectomies for benign gynaecologic problems found that increasing the surgical volume could not reduce the rate of serious complications [37]. The population-based study by Ruiz et al. demonstrated that the comparative safety of abdominal and laparoscopic hysterectomy was influenced by surgeon volume, but that the volume-outcomes relationship was not present in robotic-assisted or vaginal hysterectomy [28].

Interestingly, in ARH cohort, patients with stage IIB were less likely to be treated by high-volume or intermediate-volume surgeons. However, in LRH cohort, stage IIB disease was more frequently seen by high-volume or intermediate-volume surgeons than by low-volume surgeons. A possible explanation for this might be different guideline compliance rates in different kinds of hospitals. In the ARH cohort, 59.5% of patients treated by high-volume or intermediate-volume surgeons received treatment from a cancer centre, whereas in the LRH cohort, only 29.2% of patients treated by high-volume or intermediate-volume surgeons received treatment from cancer centre (Additional file 1: Table S2). This might be related to higher compliance with stage IIB treatment guidelines in cancer centres. Matsuo et al. had similar findings in their assessment of hospital volume. Matsuo et al. examined 5,964 women with cervical cancer who underwent RH (mostly ARH) and found that stage IIB disease was less frequently seen in the high-surgical volume group than in the other groups [10]. In our study, patients treated by high-volume or intermediate-volume surgeons were less likely to receive neoadjuvant chemotherapy in both the ARH cohort (OR = 0.81, 95% CI = 0.72–0.91) and LRH cohort (OR = 0.69, 95% CI = 0.57–0.82). A similar observation was observed by Matsuo et al. who found that women in the high-volume group were less likely to receive neoadjuvant chemotherapy before RH [10]. Adherence to guidelines by surgeons with different surgical experience merits further exploration.

Numerous factors affect surgical experience. First, surgical experience has been assessed and quantified by different metrics, such as annual surgeon volume (frequency), technique-specific volume, surgeon cumulative volume, and years of experience [14, 38, 39]. Yasunaga et al. [40] defined surgeon volume as the number of radical hysterectomies that each gynaecologist had performed as an operating surgeon over his or her professional career. Their study showed that higher surgeon volume (greater than 200 procedures) was associated with a reduced incidence of postoperative urinary disorders. However, they did not mention the surgical route of RH. Second, surgical educators recognize that skill sets may transfer between operations [41]. Modrall et al. [42] defined the aggregate annual volume per surgeon of upper gastrointestinal operations as the “surrogate volume”, including excision of oesophageal diverticulum, gastrectomy, gastroduodenectomy, and repair of diaphragmatic hernia. Among surgeons with low-volume oesophagectomy experience, increasing the volume of surrogate operations improved the outcomes observed for oesophagectomy. However, there has been little discussion about “surrogate surgery” in gynaecological cancer surgery. It was uncertain whether surrogate operative experiences, such as experiences of extrafascial hysterectomy in benign disease and cytoreductive surgery in ovarian cancer, yield improvements in outcomes for RH. Third, the characteristics of the surgeon, such as age, may have an effect on surgical experience. For example, a surgeon age of ≤ 51 and ≥ 56 years may increase short- and long-term mortality after oesophagectomy for cancer, as the highest surgical competence is achieved between 52 and 56 years of surgeon age [43]. The reason for the decreasing performance among older surgeons might be related to mental fatigue, poorer compliance with evidence-based medicine, and higher administrative positions leading to reduced surgical frequency [38].

Currently, artificial intelligence (AI) can be applied for the prediction, screening, detection of cervical cancer or precancerous lesion and predicting the prognosis of patients [44, 45]. In addition, AI has the potential to distinguish surgeon experience and to provide access to standard surgical solutions that are independent of individuals’ experience and day-to-day performance changes. Chen et al.’s study demonstrated that machine learning can accurately classify surgeon experience based on individual stitches and sub-stitches in the vesico-urethral anastomosis of a robot-assisted radical prostatectomy [46]. Saeidi et al. achieved the enhanced autonomy necessary to perform robotic laparoscopic anastomosis of the small bowel using the Smart Tissue Autonomous Robot (STAR) and they found that autonomous robotic laparoscopic surgery outperforms expert surgeons’ manual technique and robot-assisted surgery technique in terms of consistency and accuracy during laparoscopic small bowel anastomosis experiments [47].

This population-based study is the first to examine changes in the number of surgeons and annual surgeon volume for cervical cancer over time, and to explore the association between surgeon volume and complications after ARH and LRH in China. First, a strength of our study is its large sample size. We reviewed 22,684 cases treated at 42 hospitals over a 13-year period so that data on the rare adverse events could be collected. Second, we also included clinical characteristics, such as FIGO stage, histology, preoperative treatment, hysterectomy type, and lymph node dissection type which may influence the treatment outcome [23].

We also recognize several limitations to our findings. First, this study was a retrospective study in which the data were obtained from inpatient medical records or through readmission. Data on complications that may have occurred after discharge and that would have been treated by other hospitals could not be obtained. Therefore, future well-designed and prospective studies are needed to verify the work with a much larger cohort of patients. Second, we divided the patients into tertiles based on annualized surgeon volumes, but the results of the present study could not translate into clinically meaningful cut-off points. Third, although we considered a wide range of clinical characteristics and tumour characteristics, there might be other unmeasured confounding factors affecting complications, such as assistant surgeons [48], uterine size, downstream care, surgical instruments, management of complications, preoperative frailty scores [49] and subspecialty training background of each surgeon. In addition, skill transference between LRH and ARH remains unclear. Fourth, our study included only patients from 42 hospitals and the findings may not be representative of other areas in China.

## Conclusion

In the ARH cohort, the association between surgeon volume and complications for cervical cancer was complex with an increased risk of postoperative complications among intermediate-volume surgeons but the lowest postoperative complication rates for the highest-volume surgeons. In the LRH cohort, surgeon volume appeared to have no significant predictive value for complications.

## Supplementary Information

**Additional file 1. Table S1.** Multivariable analysis of factors associated with various complications. **Table S2.** The correlation between surgeon volume and hospital function in the ARH and LRH cohorts.

## Data Availability

The dataset generated and/or analysed during the current study is not publicly available due to promises of participant anonymity and confidentiality but is available from the corresponding author upon reasonable request.
