# Supplementary material for: Effect of annualized surgeon volume on major surgical complications for abdominal and laparoscopic radical hysterectomy for cervical cancer in China, 2004–2016: a retrospective cohort study
Source: BMC Womens Health. 2023 Feb 15;23:69. doi: 10.1186/s12905-023-02213-6 (PMC9933338; doi:10.1186/s12905-023-02213-6)
Supplement: Supplementary file 1 — Additional file 1. Table S1. Multivariable analysis of factors associated with various complications. Table S2. The correlation between surgeon volume and hospital function in the ARH and LRH cohorts. [file 12905_2023_2213_MOESM1_ESM.docx]

**Supplementary Table 1. Multivariable analysis of factors associated with various complications.**

|  | Abdominal surgeon volume | | | |  | Laparoscopic surgeon volume | | | |  |
| --- | --- | --- | --- | --- | --- | --- | --- | --- | --- | --- |
|  | Low | Intermediate | High |  | *P* value | Low | intermediate | High |  | *P* value |
| **Any 1 complication** | 3.06% | 3.42% | 2.01% |  | 0.10 | 5.19% | 5.69% | 5.16% |  | 0.26 |
|  |  |  | 1.20 | 0.87-1.66 | 0.27 |  |  | 1.14 | 0.82-1.58 | 0.43 |
|  |  |  | 1.39 | 1.02-1.89 | 0.04 |  |  | 1.26 | 0.95-1.67 | 0.11 |
| **Intraoperative complication** | 0.56% | 0.65% | 0.33% |  | 0.21 | 1.48% | 0.91% | 1.53% |  | 0.46 |
|  |  |  | 0.46 | 0.19-1.09 | 0.08 |  |  | 1.23 | 0.67-2.26 | 0.51 |
|  |  |  | 0.58 | 0.25-1.38 | 0.22 |  |  | 0.86 | 0.48-1.54 | 0.61 |
| Ureteral injury | 0.31% | 0.45% | 0.16% |  | 0.26 | 1.06% | 0.53% | 0.84% |  | 0.74 |
|  |  |  | 0.39 | 0.12-1.22 | 0.11 |  |  | 1.14 | 0.53-2.47 | 0.73 |
|  |  |  | 0.51 | 0.16-1.62 | 0.26 |  |  | 0.87 | 0.40-1.86 | 0.72 |
| Bladder injury | 0.06% | 0.08% | 0.02% |  | 0.14 | 0.11% | 0.15% | 0.28% |  | 0.36 |
|  |  |  | 80.72 | 0.96-6786.32 | 0.05 |  |  | 0.54 | 0.05-6.23 | 0.62 |
|  |  |  | 64.49 | 0.95-4370.79 | 0.05 |  |  | 0.22 | 0.03-1.90 | 0.17 |
| Bowel injury | 0 | 0.04% | 0 |  |  | 0.04% | 0.04% | 0.14% |  | 0.91 |
|  |  |  | —— | —— | ——^a^ |  |  | 0.46 | 0.001-27.31 | 0.71 |
|  |  |  | —— | —— | ——^a^ |  |  | 1.03 | 0.06-16.71 | 0.99 |
| Vascular injury | 0.18% | 0.04% | 0.10% |  | 0.05 | 0.15% | 0.15% | 0.24% |  | 0.92 |
|  |  |  | 0.07 | 0.007-0.75 | 0.03 |  |  | 0.90 | 0.13-6.16 | 0.91 |
|  |  |  | 0.05 | 0.004-0.65 | 0.02 |  |  | 0.73 | 0.15-3.64 | 0.70 |
| Obturator nerve injury | 0% | 0.04% | 0.04% |  |  | 0.19% | 0.04% | 0.07% |  | 0.42 |
|  |  |  | —— | —— | ——^a^ |  |  | 1.37 | 0.07-27.74 | 0.84 |
|  |  |  | —— | —— | ——^a^ |  |  | 0.16 | 0.004-5.75 | 0.31 |
| Stomach injury | 0 | 0 | 0 |  |  | 0.04% | 0 | 0 |  |  |
|  |  |  | —— | —— | ——^a^ |  |  | —— | —— | ——^a^ |
|  |  |  | —— | —— | ——^a^ |  |  | —— | —— | ——^a^ |
| **Postoperative complication** | 2.52% | 2.87% | 1.69% |  | 0.04 | 3.86% | 4.81% | 3.83% |  | 0.13 |
|  |  |  | 1.38 | 0.97-1.96 | 0.07 |  |  | 1.09 | 0.75-1.58 | 0.65 |
|  |  |  | 1.55 | 1.11-2.15 | 0.01 |  |  | 1.34 | 0.98-1.83 | 0.07 |
| Bowel obstruction | 0.95% | 1.28% | 0.61% |  | 0.07 | 0.68% | 0.68% | 0.73% |  | 0.56 |
|  |  |  | 1.27 | 0.73-2.23 | 0.40 |  |  | 1.58 | 0.67-3.76 | 0.30 |
|  |  |  | 1.75 | 1.05-2.94 | 0.03 |  |  | 1.38 | 0.65-2.94 | 0.40 |
| Pelvic hematoma | 0.02% | 0.02% | 0 |  |  | 0 | 0.08% | 0 |  |  |
|  |  |  | —— | —— | ——^a^ |  |  | —— | —— | ——^a^ |
|  |  |  | —— | —— | ——^a^ |  |  | —— | —— | ——^a^ |
| Hemorrhage | 0.06% | 0.06% | 0.02% |  | 0.80 | 0.19% | 0.11% | 0.17% |  | 0.46 |
|  |  |  | 1.86 | 0.10-35.8 | 0.68 |  |  | 2.56 | 0.58-11.41 | 0.22 |
|  |  |  | 0.78 | 0.06-9.97 | 0.85 |  |  | 1.31 | 0.25-6.89 | 0.75 |
| Vesicovaginal fistula | 0.16% | 0.18% | 0.13% |  | 0.68 | 0.72% | 0.64% | 0.84% |  | 0.89 |
|  |  |  | 0.61 | 0.16-2.36 | 0.47 |  |  | 1.07 | 0.42-2.76 | 0.88 |
|  |  |  | 0.92 | 0.26-3.29 | 0.90 |  |  | 0.91 | 0.41-2.02 | 0.81 |
| Ureterovaginal fistula | 0.19 % | 0.43 % | 0.13% |  | 0.05 | 1.02% | 1.78% | 1.08% |  | 0.13 |
|  |  |  | 2.50 | 0.74-8.45 | 0.14 |  |  | 0.88 | 0.42-1.82 | 0.72 |
|  |  |  | 3.47 | 1.27-9.53 | 0.02 |  |  | 1.44 | 0.81-2.57 | 0.22 |
| Rectovaginal fistula | 0.02% | 0.04% | 0.02% |  |  | 0.11% | 0.11% | 0.10% |  | 0.75 |
|  |  |  | —— | —— | ——^a^ |  |  | 1.98 | 0.26-15.1 | 0.51 |
|  |  |  | —— | —— | ——^a^ |  |  | 1.06 | 0.14-8.10 | 0.96 |
| Ureteral fistula | 0.06% | 0.06% | 0.02% |  | 0.45 | 0.15% | 0.08% | 0.07% |  | 0.64 |
|  |  |  | 1.34 | 0.06-29.34 | 0.85 |  |  | 3.73 | 0.18-78.93 | 0.40 |
|  |  |  | 4.26 | 0.24-74.40 | 0.32 |  |  | 2.97 | 0.26-34.11 | 0.38 |
| Venous thromboembolism | 1.05% | 0.88% | 0.77% |  | 0.51 | 1.06% | 1.44% | 0.84% |  | 0.33 |
|  |  |  | 1.61 | 0.64-2.03 | 0.60 |  |  | 0.61 | 0.30-1.25 | 0.18 |
|  |  |  | 0.89 | 0.50-1.58 | 0.70 |  |  | 0.87 | 0.47-1.63 | 0.67 |
| Chylous leakage | 0.02% | 0 | 0 |  |  | 0.11% | 0.19% | 0.10% |  | 0.99 |
|  |  |  | —— | —— | ——^a^ |  |  | 1.08 | 0.12-9.81 | 0.95 |
|  |  |  | —— | —— | ——^a^ |  |  | 1.10 | 0.19-6.33 | 0.91 |
| **Other** |  |  |  |  |  |  |  |  |  |  |
| Death | 0 | 0 | 0.04% |  |  | 0 | 0.04% | 0.03% |  |  |
|  |  |  | —— | —— | ——^a^ |  |  | —— | —— | ——^a^ |
|  |  |  | —— | —— | ——^a^ |  |  | —— | —— | ——^a^ |

1. The frequency of positive cases was too low to perform a multivariate analysis.

The middle row for each complication class was adjusted for clinical and demographic factors, including age, year of diagnosis, urban-rural distribution, hospital function, region, city scale, mode of delivery, comorbidity, FIGO stage, gross type, histological type, preoperative treatment, lymph node dissection, and hysterectomy type reported, with the odds ratio (95% CI) of low vs. high volume. The bottom row for each complication class is adjusted for the factors mentioned above, with the odds ratio (95% CI) of intermediate vs. high volume.

**Supplementary Table 2. The correlation between surgeon volume and hospital function in the ARH and LRH cohorts.**

|  | **ARH** | | | | | **LRH** | | | | |
| --- | --- | --- | --- | --- | --- | --- | --- | --- | --- | --- |
|  | **Low volume** | | **High and intermediate volume** | | **P value** | **Low volume** | | **High and intermediate volume** | | **P value** |
|  | n | % | n | % |  | n | % | n | % |  |
| **General hospital** | 3451 | 71.3 | 3534 | 36.4 | ＜0.001 | 1998 | 75.6 | 3623 | 65.8 | ＜0.001 |
| **Cancer centre** | 1088 | 22.5 | 5771 | 59.5 |  | 450 | 17.0 | 1609 | 29.2 |  |
| **W&C centre** | 298 | 6.2 | 394 | 4.1 |  | 194 | 7.3 | 274 | 5.0 |  |
